# Prolapse surgery versus vaginal pessary in women with symptomatic pelvic organ prolapse: which factors influence the choice of treatment?

**DOI:** 10.1007/s00404-019-05046-7

**Published:** 2019-01-17

**Authors:** Barbara Bodner-Adler, Klaus Bodner, Anna Stinglmeier, Oliver Kimberger, Ksenia Halpern, Heinz Koelbl, Wolfgang Umek

**Affiliations:** 1 Department of General Gynaecology and Gynaecologic Oncology, Medical University of Vienna, Währinger Gürtel 18-20, 1090 Vienna, Austria; 2 Department of Anaesthesiology, Medical University of Vienna, Vienna, Austria; 3 Outcomes Research Consortium, Cleveland, OH USA; 4 Department of Special Gynaecology and Obstetrics, Karl Landsteiner Institute, Vienna, Austria

**Keywords:** Pelvic organ prolapse, Prolapse surgery, Pessary use, Clinical parameters

## Abstract

**Objective:**

To investigate which specific clinical factors influence patients’ choice of prolapse treatment.

**Methods:**

This study includes a total of 510 cases with symptomatic pelvic organ prolapse (POP) of stage II or higher requiring prolapse treatment. Patients were divided into surgery and pessary groups according to their own choice and treatment preference. Primary outcome of interest was to define potential clinical parameters, which contribute to surgical treatment decision.

**Results:**

A total of 252/510 (49%) women decided for prolapse surgery and 258/510 (51%) cases were treated conservatively with vaginal pessary. Hypertension, COPD as well as polypharmacy were parameters, which were statistically significantly more common in the pessary group compared to the surgically managed cases (*p* <0.05). On the contrary, women undergoing prolapse surgery were significantly younger and showed more advanced POP-Q (pelvic organ prolapse quantification) stages (*p *< 0.05). Clinical factors, such as BMI (body mass index), parity, mode of delivery and postmenopausal status, did not differ between the two groups (*p *> 0.05). Multiple logistic regression analysis revealed that advanced POP-Q stage (*p *< 0.001) as well as the absence of smoking (*p *< 0.001) were independent factors associated with surgical treatment decision.

**Conclusion:**

Women, who favoured prolapse surgery, were younger and in significant better health condition (less hypertension and COPD), but showed a significantly higher POP-Q stage compared to women choosing pessary treatment. Our data indicate that women with higher POP-Q stage and non-smokers tended to decide for prolapse surgery. This information could help in clinical practice to guide patients for the best possible treatment decision and strengthen individual counselling.

## Introduction

Pelvic organ prolapse (POP) is a common condition that increases with age and affects every second elderly woman [1]. Known risk factors contributing to prolapse are childbirth, collagen abnormalities, increasing age and a chronic increase in intra-abdominal pressure [2, 3]. Around 10% of women undergo surgery at some time in their lives for the management of prolapse [4]. In general, treatment options for patients with symptomatic POP include, beside expectant management, primarily pessary placement and surgical repair [5]. On the one hand, prolapse surgery reduces pelvic floor symptoms by restoring the anatomy of the vagina and the surrounding visceral organs; on the other hand, there are significant cost implications and surgical complications can also occur [6]. Besides, pessaries have been used as conservative treatment alternatives for POP and 50% of women will choose and continue on pessary therapy as an alternative to surgery [5]. Abdool et al. as well as Lone et al. reported in their prospective studies similar improvements in both groups regarding urinary and bowel symptoms, sexual function and quality of life [7, 8]. Regarding the choice of treatment, patients’ preference plays a very important role. However, gynaecologists do not know why patients initially choose pessary over surgical or expectant management. Better understanding in this field of treatment decision could help the clinician in better counselling and guide the patient to the right individual treatment decision.

The aim of our study was to evaluate if the clinical factors influence patients’ treatment choice and to define the potential parameters for surgical therapy decision.

## Materials and methods

This study includes 510 women treated according to their preference and our investigation was conducted at the Department of General Gynaecology and Gynaecologic Oncology, Medical University of Vienna (MUV) with recruitment between January 2013 (when the POP database was initiated) and January 2018. The study was approved by the ethics committee of Medical University of Vienna (EK No. 2184/2017) and all participants gave written, informed consent. Eligible cases were women referred to our tertiary referral urogynaecology unit for dominant POP symptoms and who wished for treatment. Exclusion criteria to participate were pelvic organ prolapse < POP-Q stage II, prolapse previously treated with pessary, predominant symptoms of urinary incontinence, contraindication against operative treatment, contraindication against pessary treatment, cancer, vaginal bleeding of unclear history and unable to read and sign informed consent. Following characteristics were assessed for each patient: age, menopausal status, weight, height, blood pressure, current medication, presence of hypertension or COPD, history of POP, POP-Q stage, Oxford Scale, parity, mode of delivery, smoking and loss of urine. Body mass index (BMI) was calculated by the formula: weight (kg)/height^2^ (m). Polypharmacy was defined as the concurrent use of more than five medications by a patient. Clinical information, including follow-up data, was obtained from the database of the Department of General Gynaecology and Gynaecologic Oncology. All patient records were pseudoanonymized and de-identified prior to analysis.

A thorough history was taken and physical examination was conducted for all patients with verified POP including a standardised urogynaecologic interview and a complete vaginal and pelvic examination. This included a urogynaecologic examination to check for genital prolapse according to ICS POP-Q system and controlled provocation with 300 ml saline in the bladder according to the International Continence Society. Furthermore, physical examination included the assessment of pelvic floor strength by the Oxford Grading Scale [9, 10].

Subsequent to physical examination all women were counselled by one consultant of the core team of urogynaecology and offered prolapse treatment either with pessary placement or surgical correction. Counselling was performed in a non-directive manner, explaining and offering a patient either conservative or surgical treatment as possible treatment options. After this information patients could choose their treatment plan by themselves and were classified into two groups according to their own treatment preference: surgical group versus pessary group.

### Pessary treatment

According to the assessment of the gynaecologist, a ring, cube or donut pessary was fitted. Stage of prolapse (assessed according to POP-Q system) influenced the choice of pessary type. Pessary fitting was performed at the outpatient clinic, pessary size and type were recorded. After placement all the patients received instructions about pessary handling treatment. Local oestrogen therapy was used in combination with pessary only in postmenopausal women. Follow-up visit was planned 1 week after insertion and afterwards every 3–4 months at the outpatient clinic.

### Prolapse surgery

Surgical intervention consisted of correction of all the affected compartments and the decision which technique was used was left to the discretion of the gynaecologist. All procedures were performed under general or spinal anaesthesia and prophylactic antibiotics were given preoperatively.

### Statistical analysis

Sample size was chosen pragmatically, using data from all the available patients treated within the aforementioned timeframe. Chi square test was used for the comparison of categorical variables between the two groups and Student’s *t* test for continuous variables. The following parameters were also entered into a multivariate logistic regression analysis of treatment choice for POP: recurrence (yes, no), age, parity, BMI, smoking (yes, no), hypertension (yes, no), COPD (yes, no), POP-Q stage and mode of delivery. A *p* value < 0.05 was considered statistically significant. The SPSS system (IBM, Armonk, NY, USA; Version 25) was used for the calculations.

## Results

This study comprised a cohort of 510 patients with symptomatic POP. Among those, a total of 252/510 (49%) women chose surgery as treatment and 258/510 (51%) cases chose pessary placement. 82/510 (16%) patients presented with recurrence of prolapse and the mean duration since the first prolapse surgery was 13.6 years (± 11.16).

Median age at the recruitment time was 63 years (range 28–95) and median BMI was 27 (range 18–62). According to the valid categories, 132/510 (26%) patients were classified as obese, 206/510 (44%) showed overweight and 124/510 (24%) had a normal BMI (48 items missing). Mean Oxford Scale for a voluntary pelvic floor muscle contraction was 1.86 (± 0.80) and 226/510 (44%) participants demonstrated a loss of urine during clinical stress test at the time of first clinical visit. The leading edge of prolapse descended an average of 2.4 ± 2.7 cm beyond the introitus (range − 2 to 13). Based on POP quantification system [9], 167 cases (33%) of our study population had stage II prolapse, 262 (51%) stage III and 81 (16%) stage IV prolapse. Baseline characteristics of the study population are shown in Table 1.

**Table 1:** Mean values (SD) of patients’ characteristics in cases with POP either managed by surgery or vaginal pessary

Parameter	Surgery group *N* = 252 (100%)	Pessary group *N* = 258 (100%)	*p* value
	*n* (%) or mean (± SD)	*n* (%) or mean (± SD)	
Age (years)	61.88 (11.86)	64.48 (13.54)	0.022
Postmenopausal	206 (82%)	214 (83%)	n.s.
BMI (kg/m^2^)	27.07(4.57)	27.37(5.61)	n.s.
Hypertension	70 (28%)	74 (29%)	0.005
COPD	52 (21%)	79 (31%)	0.014
Polypharmacy	38 (15%)	45 (17%)	0.002
Parity	2.24 (1.28)	2.24 (1.49)	n.s.
POP-Q stage			0.0001
Stage II	56 (22%)	111 (43%)	
Stage III	136 (54%)	126 (49%)	
Stage IV	60 (25%)	21 (8%)	
Mode of delivery			n.s.
SVD	200 (79%)	182 (71%)	
Vaginal operative	14 (6%)	9 (3%)	
Caesarean section	7 (3%)	6 (2%)	
No delivery	3 (1%)	5 (2%)	
Missing data	28 (11%)	56 (22%)	
Smoking	12 (5%)	21(8%)	n.s

*n.s*. not significant, *p* > 0.05; *SD* standard deviation; *SVD* spontaneous vaginal delivery

### Therapy

A total of 146/252 (58%) women underwent vaginal hysterectomy combined with McCall culdoplasty and colporrhaphia anterior/posterior, 45/252 (18%) were treated with sacrospinous ligament fixation (or hysteropexy if uterus was preserved), 34/252 (13%) received sole colporrhaphy, 18/252 (7%) were treated with laparoscopic sacrocolpopexy and in 9 (4%) cases a colpocleisis was performed.

The majority of women, treated with vaginal pessary, received a ring pessary (68%). The remaining women were either treated with cube (19%) or donut pessary (13%).

### Differences between surgical group and pessary group

Hypertension, COPD as well as polypharmacy were factors, which were statistically significantly more common in the pessary group compared to the surgically managed cases (*p* < 0.0001) (Table 1). On the contrary, women undergoing prolapse surgery were significantly younger (*p* = 0.022) and showed higher POP-Q stages compared to women managed with pessaries (*p* < 0.0001). BMI, parity, mode of delivery and postmenopausal status did not differ between the two groups (*p* >0.05).

### Multiple logistic regression analysis

Multiple logistic regression analysis was conducted to define the impact of different variables on prolapse surgery decision. Surgical versus non-surgical treatment of prolapse was defined as the dependent variable. The probability of choosing surgery rather than pessary placement increases if POP-Q stage increases (OR 2.95; 95% CI 1.94–4.49; *p* < 0.001) and decreases for non-smokers (OR 0.19; 95% CI 0.12–0.32*; p* < 0.001) (Table 2).

**Table 2:** Multivariate logistic regression analysis with surgical prolapse treatment as dependent variable and clinical characteristics as independent variables

Variable	OR	95% confidence interval	*p* value
Recurrence	0.515	0.249–1.063	0.073
Age	0.995	0.971–1.020	0.709
BMI	0.990	0.936–1.047	0.719
Parity	0.876	0.715–1.075	0.205
Smoking	0.192	0.115–0.319	< 0.001*
POP-Q	2.951	1.938–4.492	< 0.001*

*OR* odds ratio

*Statistically significant, *p* < 0.05

## Discussion

Around 10% of women undergo surgery at some time in their lives for the management of prolapse [4]. Treatment options—beside expectant management—for patients with symptomatic POP include pessary fitting and surgical repair with similar improvements in both groups regarding urinary and bowel symptoms, sexual function and quality of life [5, 7, 8]. The aim of the present study was to evaluate whether specific clinical factors contribute to patients’ choice of prolapse treatment. Furthermore, we defined potential parameters for surgical treatment decision.

### Main findings

Our study showed that women who preferred pessary use suffered significantly more frequent from hypertension, COPD as well as polypharmacy. On the contrary, women who decided for prolapse surgery were significantly younger and showed more advanced POP-Q stages compared to women managed conservatively. Multiple logistic regression analysis revealed that advanced POP-Q stage and the absence of smoking remained independent factors associated with surgical prolapse repair. In summary, our data indicate that patients with higher POP-Q stage as well as non-smokers are more likely to choose for prolapse surgery.

### Comparison with literature

In general, only a few studies investigated factors influencing therapy decision in patients with POP. Coolen et al., for example, compared the functional outcomes after pessary treatment and after prolapse surgery and found no difference in prolapse domain scores after 12 months between the two groups. However, similar to our results, the authors reported that the pessary group was significantly older than the surgical group. Furthermore, their data revealed that all women in the pessary group had higher POP-Q stages in the anterior and posterior compartments but no patients with stage IV prolapse were included in the surgery group [6]. This is not in line with our results as we detected significant higher prolapse stages in the surgery group and an advanced prolapse stage remained an independent factor for patient’s surgical treatment decision.

Some authors reported that women treated either with pessary or surgery demonstrate similar improvements in urinary and bowel symptoms, sexual function and quality of life [7]. Although POP surgery may have some advantages over pessary treatment in some cases, the risk of complications is higher in the surgical group and it might be more cost intensive.

Nevertheless, patients’ treatment preference plays an important role, especially in the willingness to try a pessary. Previous studies have shown strong patients’ preference for one or the other of two interventions. Lamers et al. found that the likelihood of choosing pessary over surgery increases with increasing age [10]. Furthermore, they reported that surgery is preferred over pessary as POP-Q stage increases [11]. This is in line with our results. Interestingly, in our study population, the distribution of pessary and surgery was well balanced as 51% of our cases chose vaginal pessary and 49% decided for a surgical correction. One may hypothesise that patients’ counselling was performed in a similar way by the involved doctors. In contrast, a study performed by Wang et al. showed a clear counselling preference to surgical correction as 68% of women chose surgery and only 33% decided for pessary in the study reported by Wang et al. [12]. Furthermore, the authors found that women in the surgical group had higher BMI, age, and longer disease duration than those in the pessary group. Our results could not detect any differences regarding age, BMI, parity and mode of delivery between the two groups.

### Limitations

We are aware of the limitations of our study. Since this is not a randomised controlled trial (RCT), selection bias might play a role. One cannot completely rule out that doctors might have counselled patients differently depending on their clinical characteristics and their own preference. However, due to our counselling strategy (non-directive manner), this fact seems to have little impact on our clinical findings. Furthermore, RCT is difficult to initiate and it is important to mention that patients’ treatment preference and autonomy in treatment decision are of great significance in prolapse management. In this study, patients’ counselling was performed in a similar way by all the involved urogynaecologists and all women had the option to choose their treatment according to their own preference. Although future prospective research is still needed, this clinical study improves the scarce literature on clinical parameters that influence patients’ treatment decision.

### Summary

In our opinion, patients’ autonomy in prolapse treatment decision is of great importance and we should respect patients’ preference of therapy. Our data indicate that women with higher POP-Q stage and non-smokers tended to choose prolapse surgery. This information could help in clinical practice to guide patients for best possible treatment decision and strengthen individual counselling. Future studies will be needed to confirm our results and to define potential parameters for surgical treatment decision.
